# Beyond Targeted Newborn Screening: A Nontargeted Metabolomics Workflow to Investigate Birthweight–Metabolome Correlations

**DOI:** 10.1021/acs.analchem.4c06061

**Published:** 2025-03-18

**Authors:** Carter K. Asef, Samuel G. Moore, Charles Austin Pickens, Carlos A. Saavedra-Matiz, Joseph J. Orsini, Konstantinos Petritis, David A. Gaul, Facundo M. Fernández

**Affiliations:** † School of Chemistry and Biochemistry, Georgia Institute of Technology, Atlanta, Georgia 30332, United States; ‡ Petit Institute of Bioengineering and Bioscience, Georgia Institute of Technology, Atlanta, Georgia 30332, United States; § Division of Laboratory Sciences, National Center for Environmental Health, Centers for Disease Control and Prevention, Atlanta, Georgia 30341, United States; ∥ Newborn Screening Program, Wadsworth Center, New York State Department of Health, Albany, New York 12237, United States

## Abstract

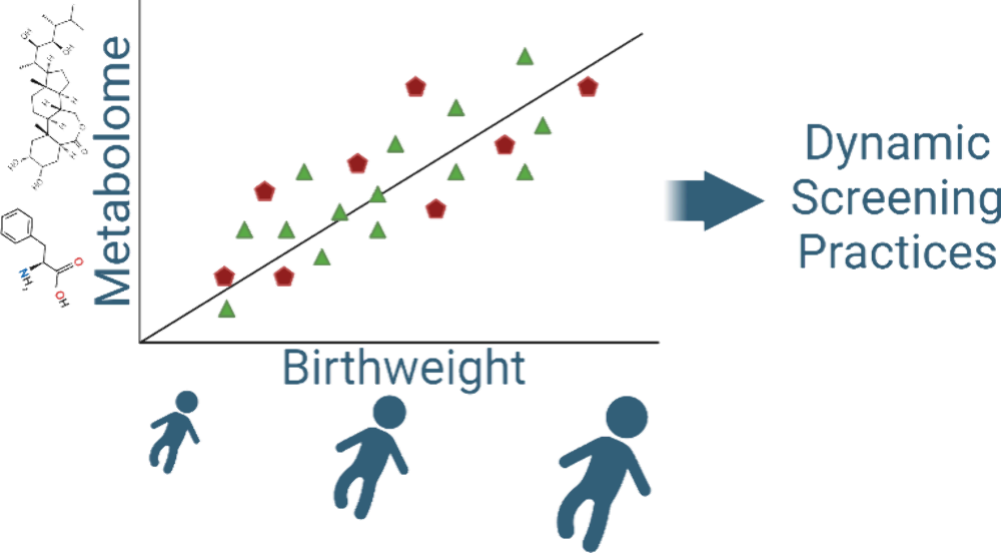

Newborn screening (NBS) is one of the United States’ largest, most successful preventative public health initiatives, improving outcomes for newborns with inborn errors of metabolism. Most disorders on the Recommended Uniform Screening Panel are screened using triple-quadrupole mass spectrometry and flow injection analysis. While these methods are sensitive and well suited for high-throughput quantitative applications, the breadth of measured analytes is limited to a relatively small number of biomarkers, which often have considerable overlaps between healthy and diseased populations. High-resolution liquid chromatography–mass spectrometry (LC–MS)-based metabolomics is now capable of profiling thousands of metabolites, making it well suited for exploratory and biomarker discovery studies. To this end, we developed a robust workflow for performing nontargeted LC–MS analysis on dried bloodspot (DBS) specimens with coverage across many metabolic pathways relevant to NBS. HILIC chromatography enabled quantitation of amino acid and acylcarnitine species while also retaining lipid species, such as lyso-phosphatidylcholines. We analyzed 810 newborn-derived DBS samples across a wide range of newborn birthweights, identifying correlations with metabolites that help to better account for the lower accuracy observed for some NBS markers (e.g., isovalerylcarnitine). Additionally, we leveraged this nontargeted workflow to capture new biomarkers and metabolic phenotypes in newborns associated with parenteral nutrition administration and maternal nicotine exposure. Two critical biomarkers were identified as useful additions to targeted screening panels: *N*-acetyltyrosine as a qualitative marker for parenteral nutrition administration and *N*-acetylputrescine as a quantitative marker for controlling birthweight variability.

## Introduction

The field of newborn screening (NBS) dates back to the 1960s with the advent of the Guthrie test for phenylketonuria (PKU).^1^ It expanded to a much wider range of disorders in the 1990s with the popularization of tandem mass spectrometry (MS/MS).^2,3^ The Newborn Screening Saves Lives Act of 2007 further facilitated metabolic testing of all newborns in the United States through the authorization of grants and expansion of the advisory committee on heritable disorders in newborns. To this date, NBS remains one of the nation’s most successful preventative public health initiatives, screening over 3 million newborns annually,^4^ avoiding developmental delay or death in approximately 1 out of 300 infants.^5^ Increasing population necessitates the highest degree of sample throughput for screening sites to keep up with birth rates. As a result, most MS-based NBS is performed using flow injection analysis (FIA) MS/MS on nominal mass triple-quadrupole mass spectrometers.^2^ While a robust platform for the rapid quantitation of target biomarkers, this approach is limited to metabolites that do not have isobaric interference in their precursor/product ion pairs.^6,7^ For some newborn disorders, the only biomarkers suitable for FIA–MS/MS analysis have considerable overlap between healthy and diseased populations, resulting in high false-positive rates to minimize the risk of missing true-positive cases. One study of isovaleric aciduria, for example, found that 99 of the 100 infants with elevated C5 acylcarnitine included in the study were false-positive results.^8^ These false positives create an unnecessary burden to clinicians who must further monitor for potential symptoms as well as cause undue stress to the families involved.

Second-tier screening is often employed to reduce false positives by conducting liquid chromatography (LC) MS/MS analysis on samples flagged during FIA–MS/MS.^9,10^ By addition of an LC separation dimension prior to MS/MS, biomarkers with better specificity may be resolved from their interferents. Methylmalonic acidemia is one such disorder where the pathognomonic marker, methylmalonic acid, is unsuitable for FIA–MS/MS as it is isomeric with the precursor/product ions from succinic acid, though these two target analytes can be quantitated independently following LC separation in a second-tier screening assay.^7^ While this is a proven approach, it greatly increases the per sample analysis time, raising cost and reporting times.

A variety of data analysis approaches have been explored as a means to further reduce false-positive rates.^11−13^ For PKU, for example, where conversion of phenylalanine to tyrosine is inhibited by the disease, the phenylalanine/tyrosine ratio has been shown to be a more effective marker than the individual metabolite concentrations.^14^ Postanalytical interpretive tools such as Collaborative Laboratory Integrated Reports (CLIR) have also been used instead of the metabolite concentrations themselves, replacing the traditional cutoff values with continuously adjusted cutoffs derived from web applications that gather information from various sources. These approaches have been shown effective for many disorders^13^ but are still limited to the relatively small number of NBS targets.

While triple-quadrupole targeted MS/MS is limited to relatively small panels of known metabolites, high-resolution MS-based metabolomics offers the ability to specifically measure the relative abundances of thousands of metabolites in a nontargeted fashion, including knowns and unknowns. Metabolomic studies are especially useful for generating new hypotheses and identifying new phenotypes of disease and, as such, much effort has been recently focused on leveraging metabolomics to improve NBS practices.^15−21^ For example, nontargeted dried blood spot (DBS) metabolomics in infants with very long-chain acylcarnitine dehydrogenase deficiency (VLCADD) was shown to improve specificity by identifying novel markers of disease.^18^ Large-scale studies, such as the one by Petrick et al.,^22^ have demonstrated the promise of nontargeted approaches to detect thousands of small molecules in extracts of archived DBS.

More recent hybrid LC–MS metabolomics approaches such as Simultaneous Quantitation and Detection (SQUAD) have opened up the possibility of performing both targeted screening and nontargeted analysis simultaneously,^23−25^ which would be ideally suited for next-generation NBS. However, the costly nature of replacing triple-quadrupole equipment with high-resolution tribrid mass spectrometers and the lower throughput of LC separations have thus far prevented wide adoption in the NBS field.

Previous research has shown birthweight to be one of the major sources of neonatal metabolome variability, including alterations to pathways of metabolites specifically targeted by NBS.^21^ Our study seeks to establish the correlation between birthweight and the newborn blood metabolome, providing a pathway for discovering more robust phenotypes of inborn errors of metabolism (IEM). To this end, we developed a robust method for conducting nontargeted LC–MS metabolomics on DBS, which includes the annotation of unknowns. The choice of chromatography chemistry was found to be of particular importance while developing this workflow as it is critical to retain the majority of the targeted NBS metabolites while also capturing a diverse set of nontargeted analytes. Using this robust and reproducible method, a large set of 810 DBS specimens was analyzed, and the quality of the obtained data was confirmed using multivariate tools. Metabolic trends associated with birthweight were identified and cross-correlated to the routinely targeted NBS markers. We also explored the ability of this metabolomics workflow to go beyond the study of birthweight effects, driving the discovery of phenotypes and biomarkers associated with maternal nicotine exposure and parenteral nutrition (PN) status.

## Materials and Methods

### Chemicals

Individual isotopically labeled standards for creatine, creatinine, and guanidinoacetate were purchased from Cambridge Isotope Laboratories (Tewksbury, MA). The NSK-A, NSK-B, and NSK-B-G1 standard mixtures containing isotopically labeled standards for many amino acids and acylcarnitine species measured in the NBS process were also purchased from Cambridge Isotope Laboratories (Tewksbury, MA). Optima-grade water and acetonitrile solvents were purchased from Fisher Scientific. LC–MS-grade ammonium formate and formic acid were purchased from MilliporeSigma.

### Sample Procurement

A cohort of 810 deidentified newborn-derived DBS specimens was assembled from samples collected by the New York State Department of Health, Wadsworth Center, with birthweights ranging from 430 to 4050 g. Ninety of these 810 samples were labeled as originating from newborns having received PN. Additionally, quality control (QC) DBS were provided by the Centers for Disease Control and Prevention (CDC) Newborn Screening and Molecular Biology Branch with validated concentrations for select amino acids and acylcarnitines.

### Sample Preparation

A working internal standard solution (WISS) was prepared by diluting all isotopically labeled internal standards into 75:25 methanol:water (v:v) to the concentrations listed in Table S1. A 3.2 mm punch from each sample was placed in individual wells of a 96-well microtiter plate. 120 μL of WISS was added to each sample well and shaken at room temperature for 1 h. 100 μL of each sample extract was transferred to a 96-well wwPTFE 0.45 μm filter plate that was washed thrice with 75:25 methanol:water (v:v) prior to use. Extracts were filtered into fresh 96-well microtiter plates using a positive pressure. Filtered extracts were transferred to individual 300 μL LC–MS vials with rubber septum caps to prevent evaporation between analytical runs. 5 μL of each sample was combined to create a pooled QC sample, which was aliquoted across 15 300 μL LC–MS vials.

### LC–MS Analysis

All samples were analyzed on a ThermoFisher Orbitrap Exploris 240 mass spectrometer coupled to a Vanquish Horizon UPLC system. C30, C18, HILIC amide, and Imtakt Intrada amino acid columns were evaluated for their retention of targeted NBS analytes, as well as for the richness of their nontargeted total ion chromatograms. A Waters BEH Amide 150 mm × 2.1 mm column with a 1.7 μm particle size was selected from these four options for its retention of all targeted amino acids and acylcarnitines while producing a richer total ion chromatogram. Solvent A consisted of 80:20 water:acetonitrile (v:v) with 10 mM ammonium formate and 0.1% formic acid; solvent B consisted of 0.1% formic acid in acetonitrile. The elution gradient started at 95% solvent B proceeding to 40% solvent B at 8 min, where it was held for 1.5 min and then re-equilibrated for 1.5 min at 95% solvent B. The column oven was held at 40 °C for the duration of the analysis. Full-scan MS data was collected for each sample at a resolution of 180,000 full-width half-maximum (fwhm) across the 70–1000 *m*/*z* range. MS/MS analysis was performed on pooled QC samples using three rounds of iterative data-dependent acquisition (DDA) (ThermoFisher AcquireX) at a resolution of 15,000 fwhm and an isolation window of 0.8 *m*/*z*. Ion activation was achieved using stepped HCD collision energies of 15, 30, and 50 V.

LC–MS run queues were initiated with the analysis of solvent blanks consisting of the solvents used in the WISS without standards as well as standard blanks consisting of WISS, which was processed through the sample extraction protocol. Four extracts from separate punches of CDC QC materials were analyzed directly after the blanks. These were repeated at the end of the queue to correct any instrumental drift quantitatively. The pooled QC sample was analyzed every 10th injection. Newborn-derived samples were split across two batches to accommodate laboratory capacity. Samples were batched after placing 3.2 mm punches into 96-well microtiter plates. Samples on the same 96-well plates were generally of similar birthweight due to the sample procurement process. Analysis of newborn-derived samples was randomized within each batch. All samples were analyzed in positive ion polarity prior to starting analysis in negative ion polarity.

### Data Analysis

Compound Discoverer 3.3 (ThermoFisher) was used to extract spectral features from the LC–MS data set by performing retention time (RT) alignment, peak picking, peak rating filtering, feature grouping, peak integration, and gap filling. Total ion chromatograms were manually reviewed to remove missed injections from the data set, with a total of 22 such injections detected. Putative identities were assigned to compounds by using mzCloud (ThermoFisher) and in-house mzVault libraries. For compounds of interest without library matches, MS/MS data was exported to a CSV file and imported into Sirius 5^26,27^ for in silico annotation. In one case where annotation could not be completed in Sirius 5, manual annotation was aided by FISh scoring within Compound Discoverer 3.3 (ThermoFisher). Differential analysis was performed using the PLS_toolbox 8.9.1 (eigenvector Research Inc.) for MATLAB (Mathworks) using autoscaling and probabilistic quotient normalization (PQN) data preprocessing steps. Cross-validation was performed with a Venetian blind width of 10% of the total data set.

When applicable, variable selection within the PLS-DA models was performed using the automatic (VIP or sRatio) method in the PLS toolbox. This method uses a variable cutoff where models are built at various VIP and sRatio levels and tested, and the model with the lowest cross-validated error (RMSECV) is preserved. Additional plots were prepared in an OriginPro 2021 (OriginLab Corporation). Quantitation was performed for features with a matched isotopic standard using the following equation: (Feature abundance/standard abundance) * standard concentration * relative response ratio. A relative response ratio of 38.71 was used to account for ∼3.1 μL of blood contained in the 3.2 mm DBS punch being diluted into 120 μL of the WISS. A full evaluation of the observed quantitative accuracy for the method, by comparison to reference values for CDC QC samples, is provided in Table S2.

## Results and Discussion

### Nontargeted Data Analysis and Potential Confounders

**Figure 1:**
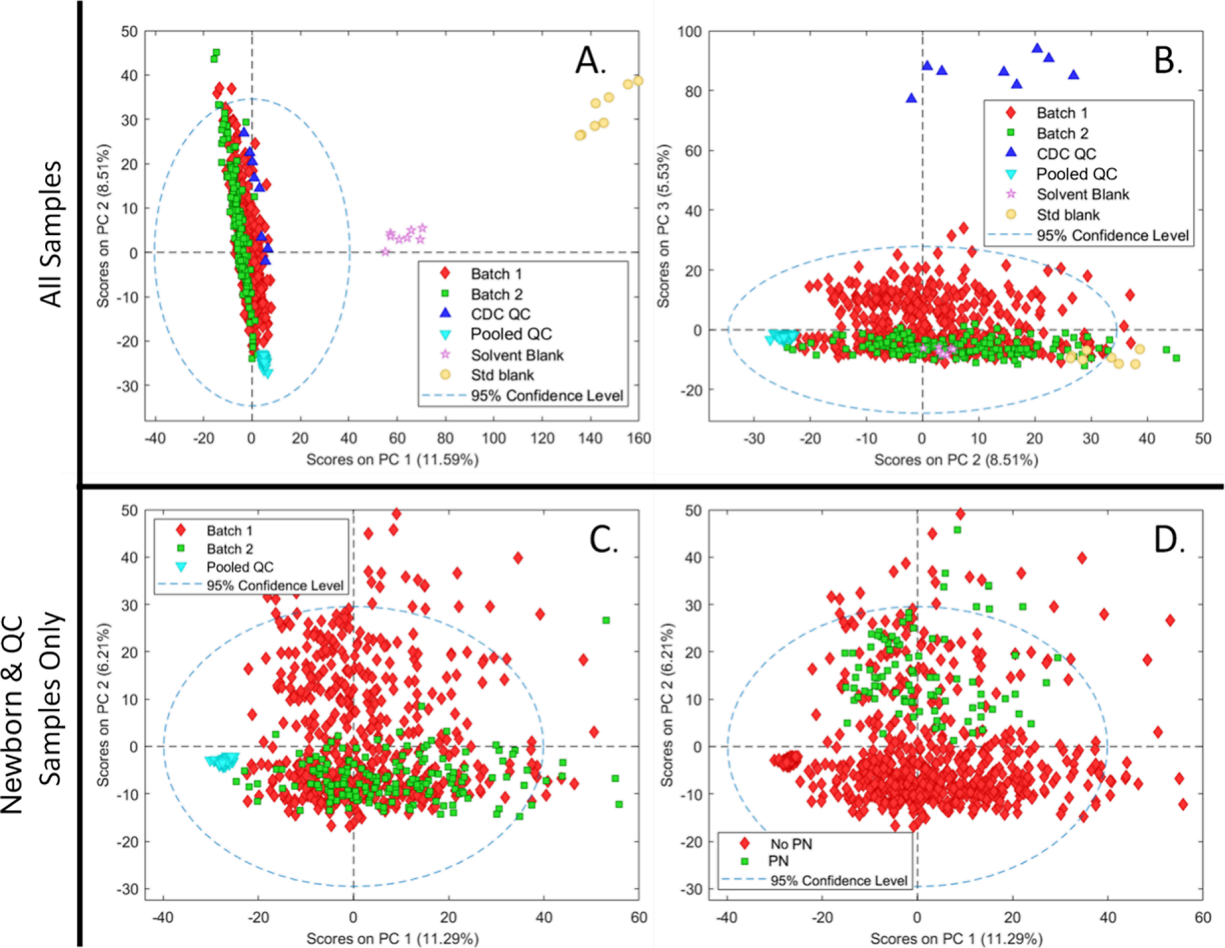
(A,B) PCA scores plots for all samples using 2339 variables. The model was also rebuilt using only newborn-derived and pooled QC samples as shown in (C) and (D). Plot C shows samples colored by batch number. Most newborn-derived samples coclustered in this plot, though a secondary cluster along PC2 could be seen, comprising only of samples from batch 1. After recoloring samples by PN status (D) and reviewing sample identities for other samples which fell into this secondary cluster, it was apparent that these samples were cases of premature birth, all of which were analyzed in batch one, the larger of the two batches.

Feature extraction and alignment of the HILIC LC–MS data set yielded 2339 (1373 positive and 966 negative) compounds following blank signal removal, with 75.6% MS/MS coverage by iterative DDA and 273 putative identifications following database searches. Principal component analysis (PCA) was performed to assess the quality of this data set (Figure 1). For a PCA model using all compounds and all samples (Figure 1A,B), the largest variation was observed between newborn-derived samples, blanks, and external CDC QC samples. These external CDC QC samples were generated by processing pooled adult donor blood, explaining the large differences observed with the newborns. The lack of outlier newborn-derived samples outside of the primary cluster indicated the successful removal of all improperly injected samples (*n* = 22).

To further examine the variance between newborn samples, a second PCA model was built containing only newborn and pooled QC samples (Figure 1C,D). Pooled QC samples were observed to cluster tightly, demonstrating the excellent technical reproducibility achieved following the QC-drift correction and PQN data normalization. While all samples from batch 2 were observed to cluster with samples from batch 1, a subset of batch 1 samples with PC2 score values trending along the vertical axis was also observed. Upon recoloring these samples by PN status and manually reviewing the remainder of the samples showing variation along PC2, it was found that they were entirely composed of low-birthweight premature birth samples. All of these samples were included in batch 1, as samples of similar birth weights were grouped in the same 96-well plate. These results hinted at the importance of both birthweight and PN status, as discussed in the next section. All full-term samples from both batches clustered together in the PC1 vs. PC2 scores plot with trends along the PC1 axis. This tight clustering, despite the large size of the cohort, demonstrated the efficacy of the QC-based instrument drift removal and batch correction.

### Metabolic Markers of PN Administration

**Figure 2:**
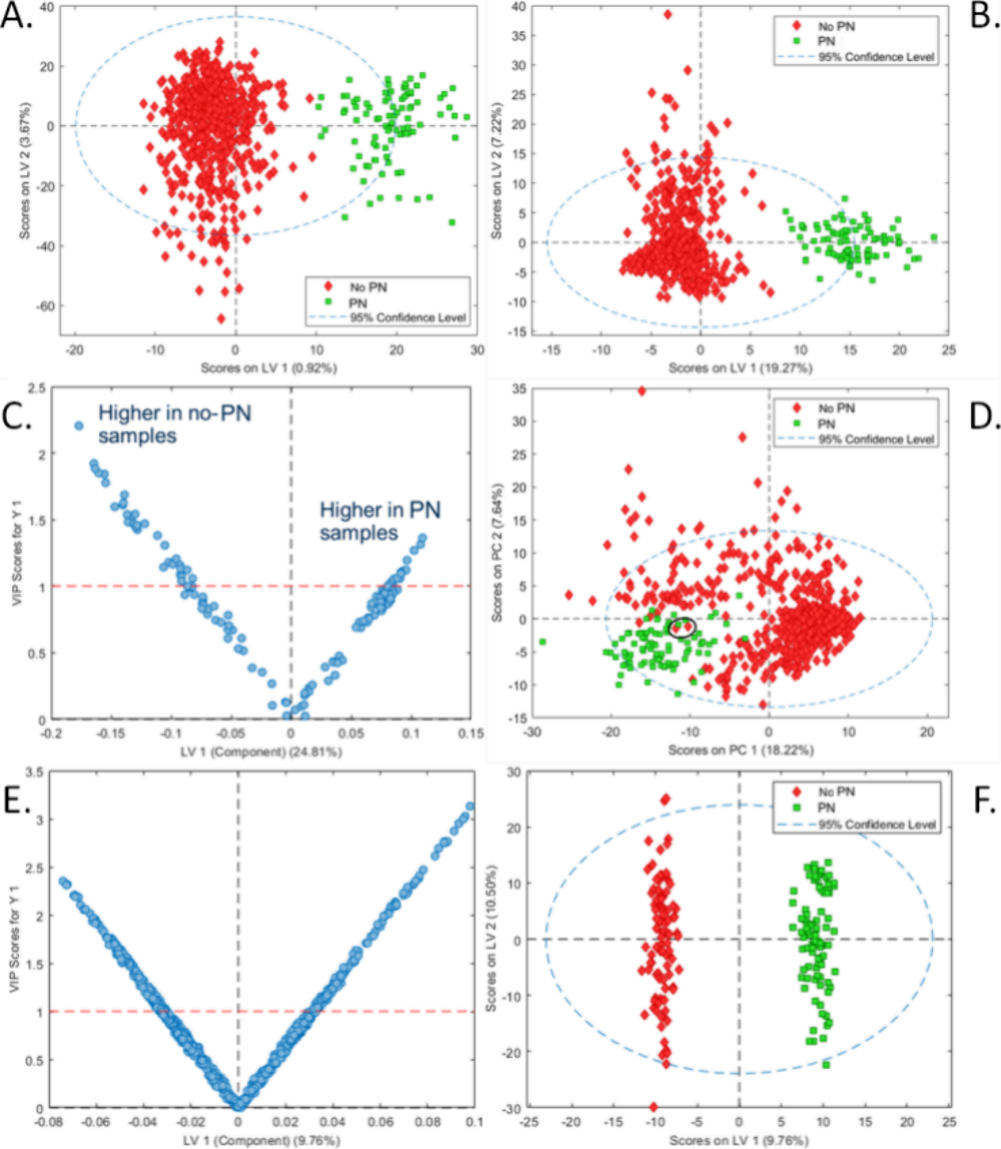
(A) Scores plot for an oPLS-DA model based on the PN status for all newborn samples using 2339 LC–MS compounds. The model was refined to use only the 389 compounds selected via automatic VIP variable selection, with the scores plot shown in (B) and the corresponding variable loadings shown in (C). These 389 variables were then used to build an additional PCA model (D) where the PN samples clearly clustered in the PC1 vs. PC2 scores plot. Notably, two samples (circled) marked at the clinic as non-PN samples coclustered with PN samples. To further validate this analysis and eliminate birthweight as a confounding factor, another oPLS-DA model was built for classifying the status of 90 PN samples against 90 non-PN samples with birthweights individually matched to the PN samples. The loading plot of this birthweight-matched model using 1007 features is shown in (E) and the corresponding scores plot in (F).

Given the differences observed between samples from premature newborns and the rest of the cohort, we sought to investigate the effect of PN administration to newborns on their metabolome. While PN is critical to the health of premature infants, this supplementation of amino acids, lipids, and other nutrients directly to the bloodstream can also confound NBS results.^28^ To examine this effect, we generated an orthogonalized partial least-squares discriminant analysis (oPLS-DA) model with all 2339 compounds extracted from the LC–MS data set by Compound Discoverer (Figure 2A). We refined this oPLS-DA model by generating a second model using only the 389 compounds following variable importance in projection (VIP) variable selection^29^ (Figure 2B). The VIP values for these 389 compounds as a function of model weights on the latent variable (LV) one are shown in Figure 2C. A list of the 50 compounds with the highest VIP values is provided in Table S3. Within the 389 compounds selected by VIP variable selection, it was noted that, although not selected in the top 50 compounds, *N*-acetyltyrosine had the highest positive PN/non-PN fold change (FC = 14.04, VIP = 1.07). Interestingly, this metabolite is the source of tyrosine used in PN mixtures as it has higher bioavailability than unmodified tyrosine.^30^*N*-Acetyltyrosine was absent in almost all non-PN samples while being elevated in many but not all PN samples. We attributed this variability to the fact that *N*-acetyltyrosine is quickly excreted following PN administration; therefore, it is likely that PN samples with low *N*-acetyltyrosine were collected several hours after administration.

**Figure 3:**
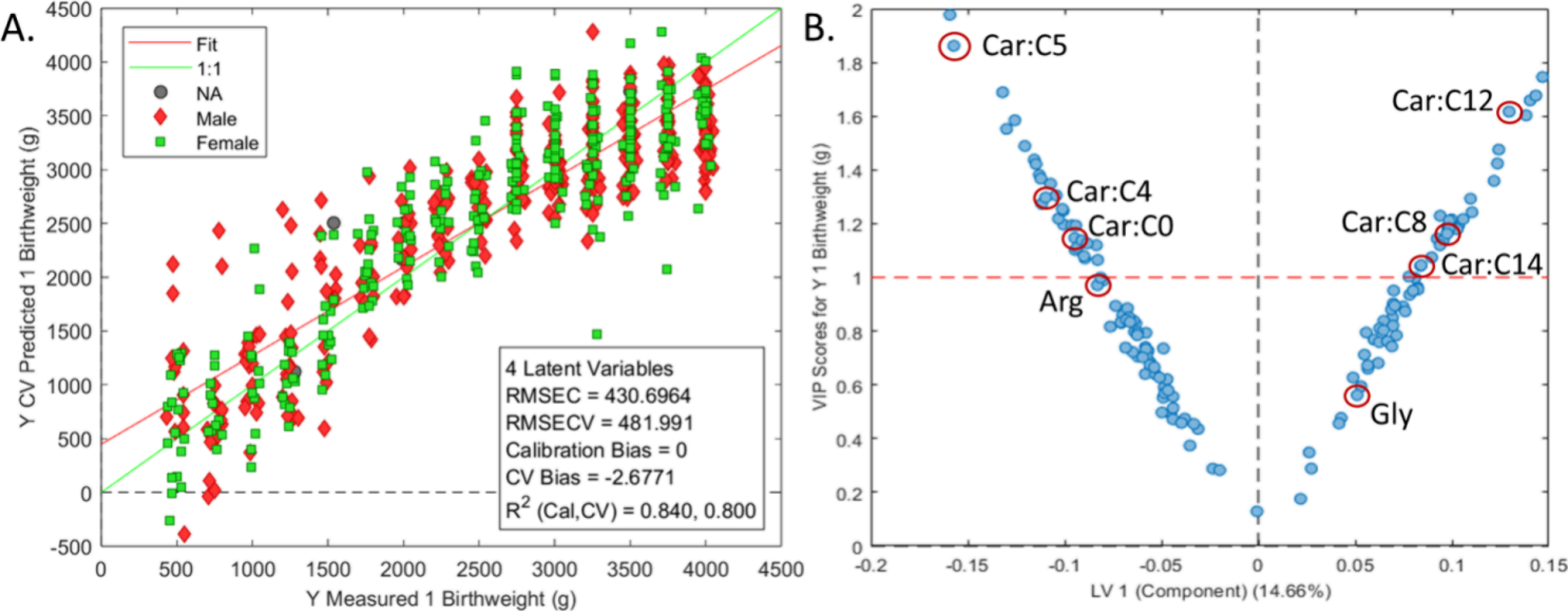
oPLS-R model for predicting birthweight using 147 putatively identified metabolites (A) and associated loadings plot (B). Metabolites positively correlated with birthweight appear in the upper right quadrant, and those negatively correlated are in the upper left quadrant. Red circles are drawn around variables corresponding to metabolites commonly targeted in NBS.

The 389 compounds selected for the refined oPLS-DA model were used to build a new PCA model of all newborn-derived samples (Figure 3D). PN samples grouped in a clearly distinct cluster, though two non-PN samples, NE58 and ND22, surprisingly coclustered with the PN samples. A review of *N*-acetyltyrosine abundance in these two samples showed they had 7367- and 65-fold higher concentrations, respectively, than the median value for all samples. These elevated levels suggested that these samples were likely mislabeled at the clinic in terms of PN status, demonstrating that *N*-acetyltyrosine may be particularly useful as a confirmatory marker for PN administration.

Ten compounds of the top 50 VIP scoring variables that had informative MS/MS spectra were imported into SIRIUS 5 for further identification, leveraging in silico structural analysis with CSI:fingerID. Not all compounds in the top 50 VIP list had MS/MS data as they were missed by the DDA algorithm. Analysis of the data from the ten compounds in SIRIUS 5 yielded two additional successful annotations: 27-carboxy-7-keto cholesterol and methylthioadenosine sulfoxide. The annotation of one of the compounds as methylthioadenosine sulfoxide was seen as likely correct, as a related metabolite, methylthioadenosine, was one of the compounds elevated in the PN cohort. In addition to methylthioadenosine and methylthioadenosine sulfoxide increasing, adenosine and methionine sulfoxide were found to decrease in the PN cohort. All of these metabolites are connected to the methionine salvage pathway, although its relevance to PN administration and/or premature birth is still unclear, although alterations to methionine metabolism have previously been observed in premature infants.^31^

*N*-Acetylputrescine was also observed as being highly elevated in the PN cohort, though this metabolite has been linked to premature birth status,^15^ making it less likely to result directly from PN administration. To better control the effects of premature birth on the metabolome, 90 non-PN samples were birth-weight-paired to samples in the PN cohort and used to build an oPLS-DA model for prematurely born infants. This model used 1007 compounds after VIP variable selection, as shown in Figure 2E,F. The top 50 compounds in terms of VIP scores are given in Table S4. A greater separation between classes was observed in this model than in that in Figure 2B, as certain markers of premature birth no longer confounded the sources of variation. One discriminant compound for PN status in this model had no candidate structures arising from in silico structural analysis, though SIRIUS 5 provided a high confidence molecular formula from the accurate mass and fragment ions. This formula yielded only one result in a search of the human metabolome database (HMDB),^32^ heptanoyl choline. SIRIUS 5 did not correctly assign this structure as a possible candidate, as it has a fixed positive charge rather than forming an [M + H]^+^ even electron ion. FISh scoring, however, assigned a confidence score of 75/100 for matching fragment ion species. This result highlighted the possibility that atypical species obscure in silico annotation efforts. Further analysis of heptanoyl choline abundance trends showed it to associate more strongly with sample plate number than with birthweight, indicating that this feature could be an artifact from the sample filtration step, or a contaminant introduced by the plate itself, despite the attention given to washing these filter plates with an extraction solvent prior to use. These findings demonstrate the importance of randomizing samples prior to extraction and batching so as to not unwittingly bias a specific sample class.

### Investigation of Metabolomic Shifts in Response to Birthweight

**Figure 4:**
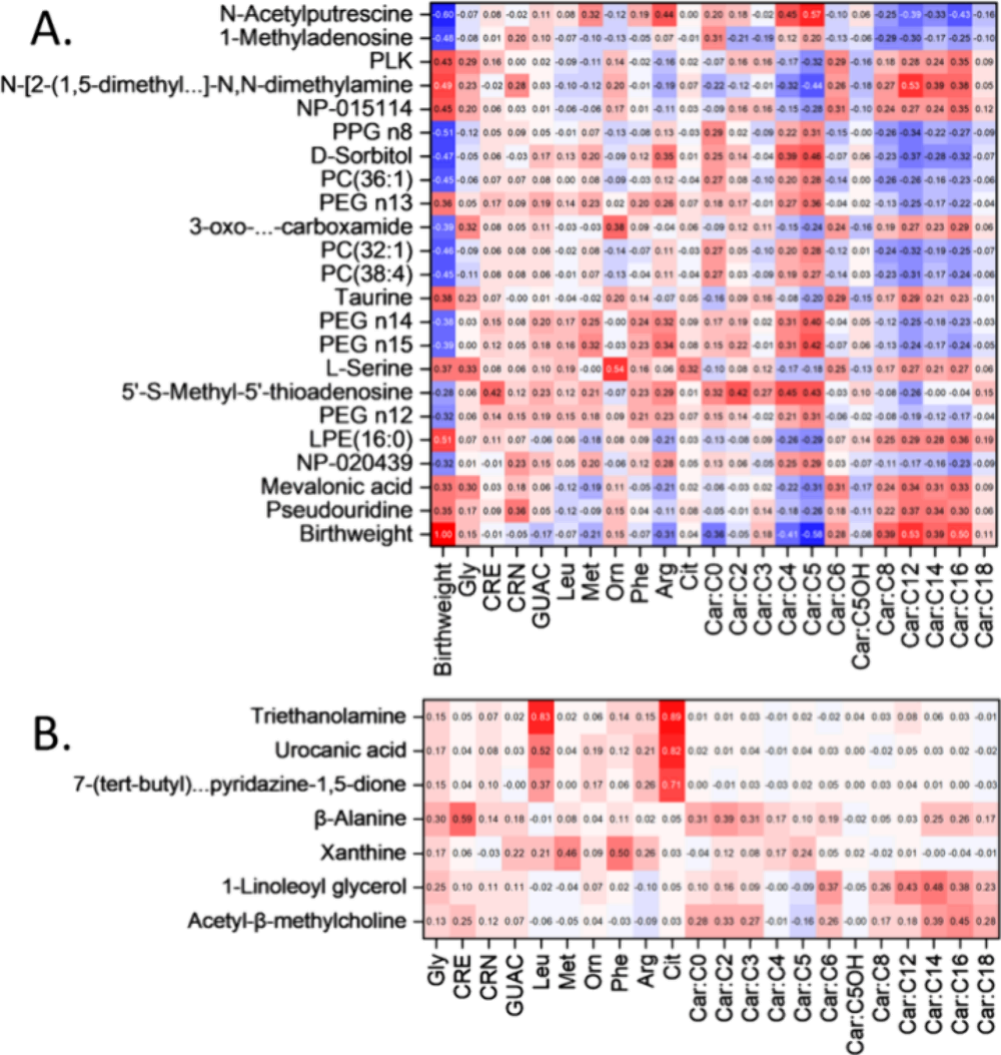
(A) Asymmetric Pearson correlation matrix for 22 named metabolites (*Y*-axis) against the quantitative values for 22 targeted metabolites commonly measured in NBS (*X*-axis). The *Y*-axis is ranked by descending VIP score in the oPLS-R model for predicting birthweight, with *N*-acetylputrescine having the highest VIP score. A correlation against birthweight is shown for both axes. (B) Asymmetric matrix for Pearson correlations of seven selected metabolites against the quantitative value for the same 22 targeted metabolites. These seven metabolites were selected as they were not used in the oPLS-R model but had the highest absolute Pearson correlations out of all named metabolites.

An orthogonalized PLS regression (oPLS-R) model was built using the 273 putatively annotated metabolites in the data set to examine associations between birthweight and the metabolome; this model was refined by VIP variable selection, leading to a set of 147 metabolites (Figure 3). Eight of the 147 metabolites are commonly targeted in the NBS process, reinforcing the notion that many of these NBS targets vary in response to birthweight and that this effect should be considered during the NBS process. The 22 highest VIP scoring metabolites from the refined oPLS-R model (excluding those targeted quantitatively in NBS screening) were compared against birthweight and against metabolites commonly quantified in NBS panels via an asymmetric Pearson correlation matrix (Figure 4A). The highest VIP score was observed for *N*-acetylputrescine, a metabolite associated with premature birth,^15^ followed by methyladenosine, which has been linked to embryonic development.^33^ Additionally, taurine was found to positively correlate with birthweight; this metabolite has been shown to be an important factor in many embryonic development pathways.^34,35^ Some other interesting trends were observed. For example, metabolites positively correlated with birthweight were also positively correlated with long-chain acylcarnitine species. Conversely, metabolites negatively correlated with birthweight were positively correlated with short-chain acylcarnitines. These acylcarnitines are part of the fatty acid metabolism and are critical for the detection of numerous IEM by NBS such as isovaleric acidemia, for which carnitine C5 is the primary NBS marker. The ratio of these birthweight-associated metabolites against NBS markers may help establish dynamic, rather than static, cutoff values and minimize false positives during NBS. For instance, C5 carnitine is known to have a significant false-positive rate when used as a singular marker for multiple diseases.^8^ Our data showed that C5 carnitine exhibited a high covariance with birthweight and *N*-acetylputrescine abundances. Thus, false positives using this specific marker may be substantially reduced by using a dynamic concentration cutoff value for different birthweight ranges or by using the C5 carnitine/*N*-acetyputrescine ratio rather than relying solely on C5 carnitine concentration. Similar trends were observed for other NBS markers such as C12, C14, and C16 carnitine, which correlated positively with birthweight and inversely with *N*-acetylputrescine.

A second Pearson correlation matrix was constructed for those annotated metabolites not selected in the 147-metabolite birthweight oPLS-R model (Figure 4B). These metabolites were ranked from top to bottom by their highest absolute Pearson correlation against NBS analytes. Three metabolites were found to correlate strongly with leucine and citrulline. Highest among these was triethanolamine, a common surfactant in personal care products. It is possible that contamination from hand cream or other personal care products used during DBS sampling may be responsible for the elevation of all five of these metabolites, highlighting the importance of glove use when gathering clinical samples and demonstrating the potential of triethanolamine as a marker for improper handling.

### Effects of Maternal Nicotine Exposure

**Figure 5:**
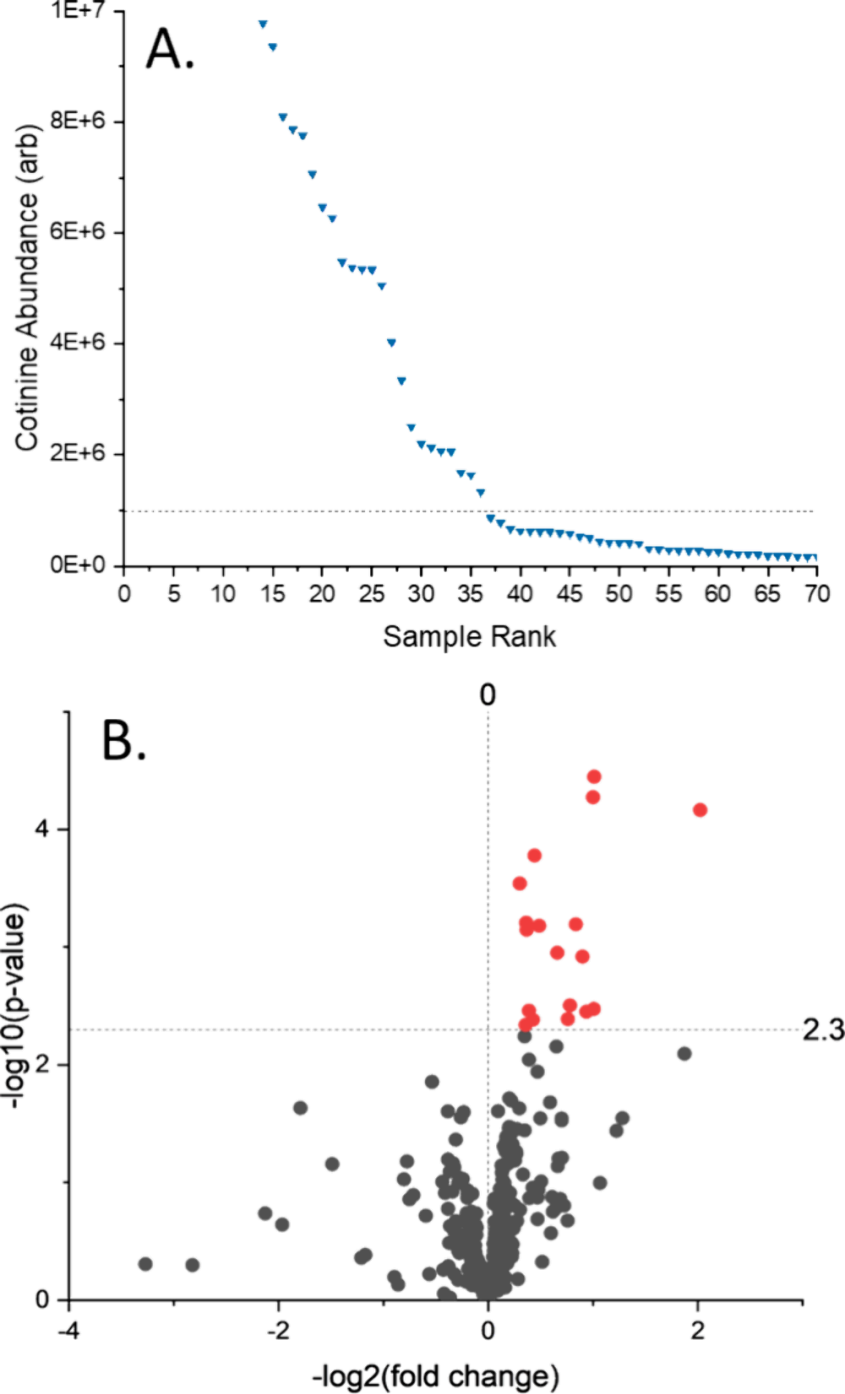
Cotinine was identified in the data set as a potential marker for nicotine exposure during pregnancy. Samples were ranked by their cotinine abundance, with abundances shown in (A). A clear trend could be observed starting at 1 × 10^7^ counts, with samples above this cutoff designated as part of the cotinine cohort. A cotinine/noncotinine volcano plot for all putatively identified features was generated as shown in (B). A −log_10_(*p*-value) of 2.3 was selected as the cutoff for significance by using the Benjamini–Hochberg corrected *p*-value for *q* = 0.05. Significantly altered metabolites are colored in red with names given in Table S5.

A high number of exogenous compounds with high VIP scores were detected in the birthweight oPLS-R model, highlighting the significance of the maternal exposome in terms of modulating the newborn metabolome. Cotinine, a primary nicotine metabolite, stood out as a key marker associated with maternal nicotine exposure. When ranking DBS samples for cotinine abundance, a clear upward trend in cotinine concentration was observed for samples with >1,000,000 counts (Figure 5A). This cutoff value was used to divide samples into two classes: a nicotine-exposed and a nonexposed group. Fold changes and *p*-values were calculated between these two groups for all putatively identified metabolites (Figure 5B). A *p*-value cutoff of 0.005 was selected following a Benjamini–Hochberg correction to yield a *q* value of 0.05, as shown in Figure 5B as a horizontal line at 2.3 after −log transformation. Significant features selected from this volcano plot are listed in Table S5. Interestingly, these included a number of common markers for NBS disorders, such as methylmalonic acid and leucine and a marker for premature birth (*N*-acetylputrescine). Well-known components of vape products (e.g., PEG, sorbitol, and vanillin) were also detected. Correlation of leucine and methylmalonic acid to cotinine demonstrates the potential for maternal nicotine exposome to directly influence the blood concentration of metabolites related to IEM, possibly confounding screening results.

## Conclusions

In this work, we present an effective workflow for the comprehensive metabolomic analysis of DBS while still maintaining high sample throughput. These methods are closely aligned with established NBS techniques to allow for a simple integration with existing NBS workflows. A robust sample extraction protocol and the LC–MS method yielded a high-quality data set with excellent coverage across a wide range of metabolites that included the amino acids and acylcarnitines commonly screened for in DBS as well as many lipid species, such as lyso-phosphatidylcholines. QC-drift correction and PQN-based normalization reduced batch effects to a minimum following 16 days of instrument analysis time. In total, 2339 deisotoped and deadducted compounds were identified above the blank levels, with highly reproducible peak shapes between samples. Many NBS targeted markers were evaluated for their quantitative accuracy, showing the potential for these methods to be integrated with future SQUAD studies. High MS/MS coverage from iterative DDA experiments led to a total of 273 annotations. PCA of the full data set revealed PN status and premature birth as critical factors in metabolic variability. Further analysis by oPLS-DA yielded a number of useful markers for these effects, such as *N*-acetyltyrosine and *N*-acetylputrescine. As PN misclassification at the clinic is a common confounder of the NBS process, *N*-acetyltyrosine may be especially useful for detecting this error during DBS screening, as demonstrated by two presumably mislabeled samples in our data set. An oPLS-R model of birthweight against 147 metabolites highlighted a number of critical pathways related to embryonic development. In silico annotation of methylthioadenosine sulfoxide reinforced the relevance of the methionine salvage pathway to premature birth. Multiple metabolites regularly quantified in the NBS process were confirmed to be highly important in this oPLS-R regression model, indicating that dynamic cutoff values for these markers of disease should help reduce false-positive rates for the detection of certain NBS disorders. Alternatively, metabolites such as *N*-acetyputrescine may be added to existing targeted screening panels and used as a ratio to control for birthweight variability of NBS markers such as C5 carnitine. We also observed correlations between markers for sample contamination, namely, triethanolamine, with some NBS analytes, such as leucine and citrulline. Further studies should be conducted to establish the interplay between these species and to ensure that NBS target analytes only originate from endogenous sources. Nontargeted NBS analysis enables the detection of unique metabolic phenotypes. In the data set presented here, markers of nicotine exposure were found to be inversely correlated with birthweight. Two NBS analytes, methylmalonic acid and leucine, were found to positively correlate with nicotine exposure and could potentially affect the NBS results.

## Supporting Information Available

Additional tables detailing the internal standards used and their concentrations, VIP scores from oPLS-DA models, evaluation of quantitative accuracy, and a list of features significantly correlated with nicotine exposure (PDF)
