# Supplementary material for: Beyond Targeted Newborn Screening: A Nontargeted Metabolomics Workflow to Investigate Birthweight–Metabolome Correlations
Source: Anal Chem. 2025 Mar 18;97(12):6563–70. doi: 10.1021/acs.analchem.4c06061 (PMC11966594; doi:10.1021/acs.analchem.4c06061)
Supplement: Supplementary file 1 — ac4c06061_si_001.pdf [file ac4c06061_si_001.pdf]

Supporting Information for:

Beyond Targeted Newborn Screening: A Non-targeted Metabolomics Workflow to Investigate Birthweight-Metabolome Correlations.

Carter K. Asef[1], Samuel G. Moore[2], Charles Austin Pickens[3], Carlos A. Saavedra-Matiz[4], Joseph J. Orsini[4], Konstantinos Petritis[3], David A. Gaul[1,2], Facundo M. Fernández[1,2*]

[1]School of Chemistry and Biochemistry, Georgia Institute of Technology, Atlanta, Georgia 30332, USA

[2]Petit Institute of Bioengineering and Bioscience, Georgia Institute of Technology, Atlanta, Georgia, 30332, USA

[3]Division of Laboratory Sciences, National Center for Environmental Health, Centers for Disease Control and Prevention, Atlanta, GA 30341, USA

[4]Newborn Screening Program, Wadsworth Center, New York State Department of Health, Albany NY, 12237, USA

Table of Contents

Table S1. Concentrations of isotopically labeled internal standards S3

Table S2. Evaluation of quantitative accuracy using QC samples..S4

Table S3. VIP scores for top 50 features from oPLS-DA model of PN status using all samples..............S5

Table S4. VIP scores for top 50 features from oPLS-DA model of PN status using only birthweight-matched samples S7

Table S5. List of features significantly correlated with nicotine exposure S9

Table S1. List of isotopically labeled internal standards used in the extraction solvent, with the related labels and final concentrations.

**Standard**	**Labels**	**Concentration (µM)**
Glycine	$^{13}C_2$, $^{15}N_1$	12.66
Alanine	D_4	2.50
Creatinine	D_3	1.00
Guanidinoacetic acid	$^{13}C_2$, $^{15}N_1$	1.00
Valine	D_8	2.50
Creatine	D_3	1.00
Ornithine	D_2	2.48
Leucine	D_3	2.46
Methionine	D_3	2.46
Car:C0	D_9	0.715
Phenylalanine	$^{13}C_6$	2.42
Citrulline	D_2	2.50
Arginine	$^{13}C_1$, D_4	2.40
Tyrosine	$^{13}C_6$	2.43
Car:C2	D_3	0.179
Car:C3	D_3	0.0375
Car:C4	D_3	0.035
Car:C5	D_9	0.0365
Car:C5OH	D_3	0.039
Car:C5DC	D_3	0.0765
Car:C8	D_3	0.0365
Argininosuccinate	$^{13}C_6$, $^{15}N_4$	2.00
Car:C12	D_9	0.037
Car:C14	D_9	0.0355
Car:C16	D_3	0.0735
Car:C16OH	D_3	0.0655
Car:C18	D_3	0.0735

Table S2. Evaluation of quantitative accuracy using CDC QC materials with certified reference concentrations for select metabolites. Four technical replicates (extracted separately) were analyzed at the start of sample analysis and again once all newborn samples had been analyzed. The drift (start *vs*. end) was determined by comparing the mean of the four replicates as measured at the start of analysis and at the end. A coefficient of variance (CV) was calculated across all eight analyses of these four technical replicates. The combined mean was used to calculate the error against the CDC reported reference value. An overall positive trend was observed as an average +38.2% error across all quantitated analytes. This positive trend was attributed to the size of the hole punch used for sampling the CDC QC materials.

Metabolite	Mean (start of analysis) (µM)	Mean (end of analysis) (µM)	Combined Mean (µM)	CV (%)	Drift start *vs.* end (%)	CDC reference concentration (µM)	Error *vs.* reference (%)
Glycine	1147.34	1110.49	1128.915	20.1	3.2	677.6	66.6
Creatine	482.32	486.02	484.1741	5.1	-0.8	357.7	35.4
Creatinine	144.28	145.07	144.673	4.0	-0.5	99.7	45.1
Guanadinoacetate	10.19	12.25	11.22173	11.7	-20.2	8.5	32.0
Leucine	377.93	366.10	372.0113	6.1	3.1	327.5	13.6
Methionine	174.93	134.15	154.5432	15.8	23.3	123.2	25.4
Ornithine	464.22	520.49	492.3549	8.6	-12.1	343	43.5
C0 Carnitine	39.58	39.61	39.5974	5.0	-0.1	38.07	4.0
Phenylalanine	348.63	329.89	339.261	6.3	5.4	297.3	14.1
Arginine	220.23	259.53	239.8802	14.0	-17.8	170.1	41.0
Citruline	137.69	143.01	140.3517	5.9	-3.9	103.9	35.1
C2 Carnitine	38.51	38.43	38.46777	4.9	0.2	26.65	44.3
C3 Carnitine	11.92	12.28	12.09958	7.9	-3.0	7.83	54.5
C4 Carnitine	3.73	3.68	3.707735	4.8	1.3	2.21	67.8
C5 Carnitine	2.24	2.01	2.12534	7.7	10.6	1.58	34.5
C5OH Carnitine	4.25	2.85	3.547408	21.8	32.9	2.48	43.0
C8 Carnitine	1.77	1.39	1.580684	14.0	21.8	0.98	61.3
C12 Carnitine	2.65	2.50	2.572128	5.8	5.8	1.85	39.0
C14 Carnitine	1.77	1.81	1.792301	8.7	-2.1	1.39	28.9
C16 Carnitine	9.12	9.06	9.089572	7.3	0.6	6.67	36.3
C18 Carnitine	3.76	4.00	3.880623	12.1	-6.4	2.81	38.1

Table S3. Putative IDs, VIP scores, and LV1 weights for the top 50 VIP score features resulting from an oPLS-DA model differentiating 90 PN samples from all non-PN samples. A positive LV1 weight indicates that a feature was more abundant in the PN cohort. Conversely, a negative LV1 weight indicates higher abundance in the non-PN cohort.

Feature Code (putative ID)	*m/z*	RT (min)	VIP Score	LV1 Weight
Pos1305 (heptanoylcholine)	216.1958	4.232	2.688847	0.13633
Pos1427	257.0574	1.507	2.366549	0.119989
Pos1366	238.1203	1.579	2.293743	0.116297
Pos2102	628.1837	1.545	2.283263	0.115766
Pos1193	181.026	1.661	2.273337	0.115263
Pos1883	480.1911	1.495	2.243546	0.113752
Neg722	431.1837	1.808	2.141054	0.108556
Pos2096	621.3103	1.402	2.117721	0.107373
Pos1979	542.2068	1.459	2.021929	0.102516
Pos2004	542.2068	1.466	2.019507	0.102393
Pos1636	335.2792	1.517	1.965878	0.099674
Pos1921	489.2271	1.433	1.946531	0.098693
Pos1952	506.2536	1.432	1.938844	0.098303
Pos1521	291.253	1.512	1.929488	0.097829
Pos2058	580.3609	4.42	1.894504	0.096055
Pos2167	686.3582	1.439	1.891654	0.095911
Pos1709	369.1695	1.449	1.83915	0.093249
Pos1856	440.3581	1.547	1.832259	0.092899
Pos1772	396.3318	1.532	1.796341	0.091078
Pos1756	386.209	1.469	1.795977	0.09106
Pos2326	972.4811	1.414	1.775706	0.090032
Pos1915	484.3844	1.559	1.737323	0.088086
Neg720 (27-carboxy-7-keto cholesterol)	429.3007	1.832	1.721709	-0.08729
Pos1775	399.193	1.454	1.720832	0.08725
Pos1710	369.1824	1.473	1.718093	0.087111
Neg238	205.0718	4.073	1.662426	-0.08429
Neg600	351.217	1.828	1.604029	-0.08133
Neg275	216.0514	3.722	1.594996	0.080869
Pos2068	594.3766	4.344	1.569549	0.079579
Pos1987	528.4107	1.571	1.564845	0.079341
Pos1121	160.108	4.584	1.540007	0.078081
Pos1049 (Methionine)	133.0318	6.343	1.522881	0.077213
Pos1423	256.0969	1.549	1.496593	0.07588
Neg666	386.0328	8.419	1.458458	0.073947
Neg567	333.2072	1.835	1.453638	-0.0737
Neg541	315.1367	1.703	1.443307	0.073179
Pos1583 (methylthioadenosine sulfoxide)	314.0917	5.402	1.423427	-0.07217

Pos1485	276.119	8.637	1.421056	0.07205
Pos1276	205.0641	2.26	1.403036	0.071137
Neg210	191.0561	4.279	1.398134	-0.07089
Pos1935	498.2827	5.731	1.383264	-0.07013
Pos1139 (Methionine sulfoxide)	166.0532	7.785	1.381655	-0.07005
Pos2111	635.3664	5.057	1.380432	0.069991
Pos2085	608.3924	4.341	1.378592	0.069897
Pos2220 PE(36:4)	740.5226	3.858	1.361795	0.069046
Pos1275	205.0641	2.661	1.357719	0.068839
Neg731	443.1239	7.692	1.348549	0.068374
Pos1308	218.1135	5.817	1.348018	-0.06835
Pos1199	183.0764	3.276	1.338342	0.067857
Neg118	164.0387	7.944	1.33058	-0.06746

Table S4. Putative IDs, VIP scores, and LV1 weights for the top 50 VIP score features resulting from an oPLS-DA model differentiating 90 PN samples from 90 birthweight-matched non-PN samples. A positive LV1 weight indicates that a feature was more abundant in the PN cohort. Conversely, a negative LV1 weight indicates higher abundance in the non-PN cohort.

Feature Code (putative ID)	*m/z*	RT (min)	VIP Score	LV1 Weight
Pos1305 (heptanolycholine)	216.1958	4.232	3.130731	0.098658
Pos1921	489.2271	1.433	3.026796	0.095382
Pos1952	506.2536	1.432	2.995714	0.094403
Pos1709	369.1695	1.449	2.950732	0.092985
Pos1636	335.2792	1.517	2.872816	0.09053
Pos1883	480.1911	1.495	2.765295	0.087142
Neg722	431.1837	1.808	2.764425	0.087114
Pos1772	396.3318	1.532	2.760505	0.086991
Pos2102	628.1837	1.545	2.754983	0.086817
Pos1521	291.253	1.512	2.749756	0.086652
Pos1427	257.0574	1.507	2.69481	0.084921
Pos1856	440.3581	1.547	2.614382	0.082386
Pos1193	181.026	1.661	2.469219	0.077812
Pos1915	484.3844	1.559	2.409298	0.075923
Pos1710	369.1824	1.473	2.377923	0.074935
Pos2167	686.3582	1.439	2.357098	0.074278
Neg238	205.0718	4.073	2.351357	-0.0741
Pos1775	399.193	1.454	2.331254	0.073464
Neg600	351.217	1.828	2.319155	-0.07308
Pos1756	386.209	1.469	2.316165	0.072989
Neg720 (27-carboxy-7-keto cholestrerol)	429.3007	1.832	2.312256	-0.07287
Pos2111	635.3664	5.057	2.290909	0.072193
Pos1979	542.2068	1.459	2.249908	0.070901
Pos2096	621.3103	1.402	2.230391	0.070286
Pos2004	542.2068	1.466	2.221056	0.069991
Neg550	321.2435	1.768	2.20813	-0.06958
Pos1423	256.0969	1.549	2.206984	0.069548
Neg569	335.2229	1.809	2.205294	-0.06949
Pos1583 (methylthioadenosine sulfoxide)	314.0917	5.402	2.1909	-0.06904
Neg606 (15(R),19(R)-Hydroxy prostaglandin F1α)	353.2326	1.826	2.186688	-0.06891
Pos1366	238.1203	1.579	2.136743	0.067334
Pos1885	464.1961	1.437	2.125294	0.066974
Pos2326	972.4811	1.414	2.107519	0.066414
Neg87	145.087	2.136	2.105487	-0.06635
Pos1987	528.4107	1.571	2.099878	0.066173
Neg541	315.1367	1.703	2.091031	0.065894

Pos1121	160.108	4.584	2.090236	0.065869
Neg570	337.2385	1.822	2.058936	-0.06488
Neg210	191.0561	4.279	2.055713	-0.06478
Neg275	216.0514	3.722	2.047484	0.064522
Neg567	333.2072	1.835	2.014492	-0.06348
Pos1867	447.1695	1.437	2.012834	0.06343
Neg537	313.1293	1.701	1.994123	0.06284
Pos2058	580.3609	4.42	1.98815	0.062652
Pos1591	317.2111	1.617	1.968909	-0.06205
Pos1461 (adenosine)	268.1041	4.561	1.955354	-0.06162
Pos1550	301.2162	1.616	1.937181	-0.06105
Pos1199	183.0764	3.276	1.931948	0.060881
Pos1635 (Bicyclo prostaglandin E2)	335.2218	1.62	1.924683	-0.06065
Pos1935	498.2827	5.731	1.914265	-0.06032

Table S5. List of putatively identified metabolites significantly altered by nicotine exposure with their Log_2 fold changes and associated p-values.

**Metabolite**	**Log_2 fold change**	**p-value**
D-Sorbitol	0.999	5.3E-05
Triethanolamine	2.022	6.8E-05
Leucine	0.441	1.7E-04
Methylmalonic acid	0.302	2.9E-04
Trigonelline	0.362	6.2E-04
PEG n14	0.836	6.4E-04
1-methyladenosine	0.487	6.6E-04
NP-007257	0.659	1.1E-03
PEG n15	0.899	1.2E-03
PEG n13	0.780	3.1E-03
PEG n16	1.007	3.3E-03
N-acetylputrescine	0.390	3.5E-03
4-hydroxy-3-methoxyphenylglycol sulfate	0.426	4.1E-03
Dihydrothymine	0.358	4.6E-03
